# Study on the diagnostic and differential value of thalassemia through combined blood routine examination and reticulocyte detection

**DOI:** 10.3389/fped.2025.1616766

**Published:** 2025-07-29

**Authors:** Haiyan Ke, Hongxia Zhang, Hong Jiang, Sheng Li, Hui Wang

**Affiliations:** ^1^ Department of Clinical Laboratory, Huangshi Maternity and Children’s Health Hospital, Affiliated Maternity and Children’s Health Hospital of Hubei Polytechnic University, Huangshi, Hubei, China; ^2^ Huangshi Key Laboratory of Birth Defects Prevention, Huangshi, Hubei, China

**Keywords:** complete blood count, differential diagnosis, logistic regression models, reticulocyte detection, thalassemia

## Abstract

**Objective:**

This study aims to explore the diagnostic and differential values of thalassemia genotypes using combined complete blood count (CBC) and reticulocyte parameter analysis in neonates, considering physiological age-related hematological changes.

**Methods:**

A retrospective study was conducted from October 2023 to March 2024 involving 315 neonates in the Huangshi area who underwent thalassemia gene detection, CBC, and reticulocyte analysis. Participants were categorized into a control group (*n* = 83), α-thalassemia group (*n* = 177), and β-thalassemia group (*n* = 55). Further subgrouping was based on genotype severity and age (≤28 days and >28 days). A comparative analysis of hematological parameters was performed, and logistic regression models were developed to predict and differentiate thalassemia types.

**Results:**

In both age strata, the α-thalassemia group exhibited significantly higher red blood cell (RBC) counts but lower mean corpuscular volume (MCV), mean corpuscular hemoglobin (MCH), and mean corpuscular hemoglobin concentration (MCHC) compared to control and β-thalassemia groups (*P* < 0.05). The β-thalassemia group showed significantly higher red cell distribution width (RDW) than the other groups (*P* < 0.05). Reticulocyte parameters also showed distinct patterns: the α-thalassemia group had lower reticulocyte counts (RET#), while the β-thalassemia group had a higher immature reticulocyte fraction (IRF). A logistic regression model combining RBC, MCHC, RET#, and IRF to differentiate α- from β-thalassemia achieved an area under the curve (AUC) of 0.879, with a sensitivity of 72.7% and specificity of 89.2%. The combined models generally outperformed single-parameter analyses.

**Conclusions:**

Hematological parameters like MCV, MCH, and RDW are effective initial screening indicators for neonatal thalassemia. Integrating reticulocyte analysis with routine CBC enhances screening accuracy and aids in the differential diagnosis of α- and β-thalassemia. This combined, age-stratified approach is valuable for improving early detection and management strategies.

## 1 Introduction

Thalassemia is a hereditary hemolytic anemia caused by mutations or deletions in the globin genes, which impair the synthesis of hemoglobin. This condition is characterized by reduced or absent hemoglobin production, leading to a range of clinical manifestations depending on the severity of the genetic defect. Thalassemia is a global public health concern, with significant prevalence in regions like Southeast Asia, the Mediterranean, and the Middle East (1–4). It is one of the most common inherited blood disorders and remains a major cause of morbidity and mortality in affected populations.

Thalassemia is divided into two main types based on the affected globin chain: α-thalassemia and β-thalassemia. The severity of the disease can vary greatly, ranging from asymptomatic or mildly symptomatic cases to life-threatening forms. Mild thalassemia may not require treatment and may be detected incidentally during routine blood tests. Moderate to severe forms, however, require regular blood transfusions, iron chelation therapy, and can have a poor long-term prognosis (5–7).

The diagnosis of thalassemia traditionally relies on genetic testing, which identifies mutations or deletions in the α or β globin genes. However, in many areas, especially in developing countries, genetic testing may not be readily available due to high costs and limited healthcare infrastructure. As such, diagnosing and differentiating between thalassemia types using routine hematological examinations and reticulocyte measurements is of critical importance, especially for early detection and management.

While complete blood count (CBC) is commonly used to assess the general health of individuals, it can also provide valuable insights into the presence and severity of thalassemia. Key parameters such as mean corpuscular volume (MCV), mean corpuscular hemoglobin (MCH), red cell distribution width (RDW), and hemoglobin concentration often exhibit characteristic changes in thalassemia patients (8). In addition, reticulocyte counts and indices—markers of new red blood cell production—can help differentiate between thalassemia types, as thalassemia is typically associated with an increased reticulocyte count due to compensatory hemolysis (9).

In recent years, there has been growing interest in logistic regression models that combine multiple hematological markers to improve the accuracy of diagnosis and differentiation of thalassemia subtypes. These models can be particularly useful when genetic testing is unavailable or when there is a need for quick and reliable screening methods in large populations.

This study was conducted to investigate the diagnostic value of combined CBC and reticulocyte detection in thalassemia. We aimed to explore how these markers can help not only in the early detection of thalassemia but also in differentiating between various thalassemia genotypes and phenotypes, which is crucial for the proper management and treatment of affected individuals.

## 2 Methods and materials

### 2.1 Clinical data

A total of 15,805 neonatal heel blood samples were collected from newborns born between from October 2023 and March 2024 at Huangshi Maternal and Child Health Hospital and 57 collaborating institutions at various levels across Huangshi City, including county and township areas. Neonates with preliminary positive results from hemoglobin electrophoresis screening were recalled for further thalassemia gene detection, routine blood tests, and reticulocyte counts. A total of 315 subjects were included in the study. One case of αβ-thalassemia was identified but was excluded from the comparative statistical analyses due to the sample size being insufficient for meaningful comparison, making the final analytical cohort 315 subjects. Based on genetic deletions and mutation types, the study participants were divided into the following groups: control group (*n* = 83), α-thalassemia group (*n* = 177), and β-thalassemia group (*n* = 55). The α-thalassemia group was further subdivided into α-stationary type (*n* = 82), α-mild type (*n* = 94) and one case of α-intermediate type (*n* = 1) while the β-thalassemia group was divided into β-mild type (*n* = 49) and β-moderate type (*n* = 6). This study was approved by the Ethics Committee of Huangshi Maternal and Child Health Hospital (2024-LWSC-006), and informed consent forms were waived due to the retrospective nature of the research and the use of anonymized data.

### 2.2 Inclusion and exclusion criteria

Inclusion Criteria: Neonates with preliminary positive results from hemoglobin electrophoresis screening, who were recalled for thalassemia gene detection, routine blood tests, and reticulocyte counts, and who participated in all these programs, were included in the study.

Exclusion Criteria: Neonates who did not participate in all of the aforementioned programs were excluded from the study.

### 2.3 Methods

Heel blood samples were collected from neonates and prepared as dried blood spots. Hemoglobin electrophoresis experiments were conducted using capillary electrophoresis instruments (model: CAPILLARYS2) and matching reagents produced by the French company SEBIA. Neonates with preliminary positive results in the hemoglobin electrophoresis screening were recalled for further examination. According to the Sebia criteria, the following were considered positive results for primary screening for thalassemia based on hemoglobin electrophoresis:

Hb Bart's > 0
HbE > 0
HbA ≤ 7.3
7.3 < HbA ≤ 11.5 and HbA2 > 0
11.5 < HbA ≤ 15 and HbA2/HbA ratio ≥ 0.015
15 < HbA ≤ 20 and HbA2/HbA ratio ≥ 0.020
HbA > 20 and HbA2/HbA ratio ≥ 0.025

For blood routine examination and reticulocyte detection, 2 ml venous blood samples were collected using EDTA-K2 anticoagulant vacuum blood collection tubes. The Mindray BC6800 (Shenzhen Mindray Biomedical Electronics Co., Ltd.) fully automated hematology analyzer, along with its original matching reagents, was used for the analysis. Another 2 ml venous blood sample was collected for α-globin gene analysis via gap-polymerase chain reaction (Gap-PCR) to detect three common deletions (–SEA, −α^3.7^, and −α^4.2^). For β-globin gene analysis PCR combined with reverse dot blot hybridization was used to detect 17 common mutation sites, with the most prevalent in our cohort being IVS-II-654 (C>T), followed by CD17 (AAG>TAG) and CD41/42 (-TTCT).

### 2.4 Criteria for grouping

Thalassemias were classified into α-thalassemia and β-thalassemia based on the type of gene deletions and mutations. α-Thalassemia was further categorized according to the number of α-globin gene deletions: α-stationary type (deletion of one alpha chain), α-thalassemia mild type (deletion of two alpha chains), α-thalassemia moderate type (deletion of three alpha chains), and α-thalassemia severe type (deletion of four alpha chains). β-Thalassemia was classified into mild, moderate, and severe types based on gene mutations and clinical manifestations (6).

According to the pediatric anemia diagnostic criteria established by the National Pediatric Hematology Conference in China, neonates with hemoglobin (Hb) levels of less than 145 g/L for those ≤28 days old, and less than 90 g/L for those aged 1–4 months, can be diagnosed with anemia (10). Diagnostic criteria for microcytic hypochromic anemia included: mean corpuscular volume (MCV) less than 80 fL, mean corpuscular hemoglobin (MCH) less than 27 pg, and mean corpuscular hemoglobin concentration (MCHC) less than 320 g/L (11).

### 2.5 Observation indicators

The following blood routine examination results were observed and compared: RBC count, hemoglobin (Hb), hematocrit (HCT), MCV, MCH, MCHC, red cell distribution width (RDW), reticulocyte percentage (RET%), reticulocyte count (RET#), immature reticulocyte fraction (IRF), low fluorescence reticulocytes (LFR), medium fluorescence reticulocytes (MFR), and high fluorescence reticulocytes (HFR).

### 2.6 Statistical analysis

All data were collected using Microsoft Excel 2010 and analyzed using SPSS 20.0 statistical software. Quantitative data are expressed as mean ± standard deviation, and qualitative data are presented as frequency and proportion. To account for physiological changes in hematological parameters during the neonatal period, participants were further stratified into two age groups for analysis: those aged ≤28 days (*n* = 151) and those aged >28 days (*n* = 164). Inter-group comparisons were performed using one-way analysis of variance (ANOVA), and pairwise comparisons were conducted using the Student-Newman-Keuls (S-N-K) method. In cases where variances were not homogeneous, Dunnett's *t*-test was used. Logistic regression models were established to predict thalassemia. The first logistic regression model, which included 260 samples from the control and α-thalassemia groups, used HCT, MCH, and RET% as independent variables to predict α-thalassemia. The second model, involving 138 samples from the control and β-thalassemia groups, used MCV, RDW, and MFR as independent variables to predict β-thalassemia. The third logistic regression model, including 232 samples from the α-thalassemia and β-thalassemia groups, used RBC, MCHC, RET#, and IRF as independent variables to differentiate between α-thalassemia and β-thalassemia. All models employed stepwise regression, with a *p*-value of 0.1 set for the inclusion and exclusion of independent variables. Receiver operating characteristic (ROC) curves were constructed, the area under the curve (AUC) was calculated, and the cut-off values were determined. All statistical tests were performed using two-tailed tests, with *P* < 0.05 considered to indicate statistically significant differences.

## 3 Results

### 3.1 General data

A total of 315 newborns with positive initial screening results were included in the study, comprising 182 males and 133 females. The average gestational age was 38.4 ± 1.9 weeks, the average age was 28.9 ± 19.1 days, and the average birth weight was 3,252 ± 529 g. No statistically significant differences were observed in terms of gender, age, or weight among the three groups (*P* > 0.05).

### 3.2 Comparison of blood routine examination parameters between thalassemia patients and controls

After stratifying by age, the analysis showed consistent trends within both the ≤28 days and >28 days age groups. In both cohorts, the α-thalassemia group exhibited significantly increased RBC count and HCT compared with the control and β-thalassemia groups (*P* < 0.05), while MCV, MCH, and MCHC were significantly lower (*P* < 0.05). The RDW in the β-thalassemia group was significantly higher than that in both the control and α-thalassemia groups across both age strata (*P* < 0.05). In the >28 days group, MCV and MCH in the β-thalassemia group were significantly lower than in the control group (*P* < 0.05) (Table 1).

**Table 1:** Comparison of blood routine examination parameters among different thalassemia phenotypes and control groups, stratified by age.

Index	Age Group	Control group	α-thalassemia group	β-thalassemia group	F	*P*
RBC (10^12^/L)	≤28 days (*n* = 40, 85, 26)	4.15 ± 0.80	4.70 ± 0.82^a^	4.10 ± 0.60^b^	21.551	<0.001
	>28 days (*n* = 43, 92, 29)	3.85 ± 0.75	4.45 ± 0.77^a^	3.88 ± 0.55^b^	22.618	<0.001
HB (g/L)	≤28 days	124.50 ± 22.10	127.91 ± 24.15	120.33 ± 23.50	2.880	0.060
	>28 days	116.30 ± 21.50	119.98 ± 23.50	113.31 ± 22.60	2.510	0.084
HCT (%)	≤28 days	38.91 ± 7.10	41.05 ± 7.60^a^	37.95 ± 7.21	6.992	0.001
	>28 days	36.00 ± 6.80	38.20 ± 7.30^a^	34.80 ± 6.90^b^	7.010	0.001
MCV (fL)	≤28 days	98.50 ± 9.10	89.51 ± 9.01^a^	93.54 ± 9.80^b^	21.871	<0.001
	>28 days	91.80 ± 8.80	84.72 ± 8.70^a^	88.81 ± 9.60^a^^,^^b^	21.007	<0.001
MCH (pg)	≤28 days	31.10 ± 3.21	27.99 ± 3.20^a^	30.15 ± 3.50^b^	26.543	<0.001
	>28 days	29.62 ± 3.10	26.39 ± 3.15^a^	28.52 ± 3.45^a^^,^^b^	27.260	<0.001
MCHC (g/L)	≤28 days	321.35 ± 13.20	313.50 ± 11.90^a^	323.80 ± 11.00^b^	18.012	<0.001
	>28 days	317.22 ± 13.10	310.21 ± 11.70^a^	319.45 ± 10.85^b^	18.341	<0.001
RDW (%)	≤28 days	19.01 ± 11.01	22.10 ± 11.55	25.99 ± 14.50^a^^,^^b^	30.115	<0.001
	>28 days	18.53 ± 10.80	21.65 ± 11.30	25.05 ± 14.30^a^^,^^b^	29.120	<0.001

RBC, red blood cell; HB, hemoglobin; HCT, hematokrit; MCV, mean corpuscular volume; MCH, mean corpuscular volume; MCHC, mean corpuscular hemoglobin concentration; RDW, red cell distribution width.

^a^ Compared with the control group, *P* < 0.05.

^b^ Compared with the α-thalassemia group, *P* < 0.05.

Subgroup analysis further clarified these findings. In both age groups, the α-thalassemia mild type group had the highest RBC counts. In neonates ≤28 days, the α-thalassemia stationary type group had significantly higher Hb levels compared to both the control and α-mild type groups (*P* < 0.05). Consistently, the α-mild type group exhibited the lowest MCV, MCH, and MCHC values compared to both the control and α-stationary type groups in both age cohorts (*P* < 0.05) (Table 2). Additionally, the β-thalassemia mild type group had a significantly higher RDW compared to the control group in both age strata (*P* < 0.05). A similar trend was observed when comparing the β-mild type to the β-moderate type, though it did not reach statistical significance, likely due to the small sample size of the moderate group (Table 3).

**Table 2:** Comparison of blood routine examination parameters among different α-thalassemia subgroups and control group, stratified by age.

Index	Age Group	Control group	α-thalassemia silent type group	α-thalassemia mild type group	F	*P*
RBC (10^12^/L)	≤28 days (*n* = 40, 40, 45)	4.15 ± 0.80	4.41 ± 0.70^a^	4.96 ± 0.85^a^^,^^b^	14.112	<0.001
	>28 days (*n* = 43, 42, 49)	3.85 ± 0.75	4.17 ± 0.65^a^	4.70 ± 0.79^a^^,^^b^	13.544	<0.001
HB (g/L)	≤28 days	124.50 ± 22.10	131.50 ± 22.50^a^	124.65 ± 24.50^b^	2.105	0.126
	>28 days	116.30 ± 21.50	125.60 ± 22.00^a^	115.10 ± 23.80^b^	3.201	0.044
HCT (%)	≤28 days	38.91 ± 7.10	41.50 ± 7.00	40.65 ± 7.80	1.545	0.217
	>28 days	36.00 ± 6.80	39.20 ± 6.85^a^	37.30 ± 7.65	1.503	0.226
MCV (fL)	≤28 days	98.50 ± 9.10	96.80 ± 5.40	83.20 ± 5.70^a^^,^^b^	63.145	<0.001
	>28 days	91.80 ± 8.80	92.10 ± 5.25	78.90 ± 5.55^a^^,^^b^	58.892	<0.001
MCH (pg)	≤28 days	31.10 ± 3.21	30.70 ± 1.70	25.60 ± 1.80^a^^,^^b^	85.221	<0.001
	>28 days	29.62 ± 3.10	29.25 ± 1.68	24.09 ± 1.72^a^^,^^b^	77.421	<0.001
MCHC (g/L)	≤28 days	321.35 ± 13.20	319.40 ± 11.20	308.25 ± 10.10^a^^,^^b^	16.002	<0.001
	>28 days	317.22 ± 13.10	315.98 ± 11.05	305.61 ± 9.88^a^^,^^b^	14.949	<0.001
RDW (%)	≤28 days	19.01 ± 11.01	21.60 ± 12.40	22.55 ± 10.80	1.258	0.288
	>28 days	18.53 ± 10.80	21.08 ± 12.25	22.15 ± 10.55	1.115	0.331

RBC, red blood cell; HB, hemoglobin; HCT, hematokrit; MCV, mean corpuscular volume; MCH, mean corpuscular volume; MCHC, mean corpuscular hemoglobin concentration; RDW, red cell distribution width.

^a^ Compared with the control group, *P* < 0.05.

^b^ Compared with the α-thalassemia silent type group, *P* < 0.05.

**Table 3:** Comparison of blood routine examination parameters among different β-thalassemia subgroups and control group, stratified by age.

Index	Age Group	Control group	β-thalassemia moderate type group	β-thalassemia mild type group	F	*P*
RBC (10^12^/L)	≤28 days (*n* = 40, 3, 23)	4.15 ± 0.80	3.95 ± 0.40	4.12 ± 0.62	0.255	0.776
	>28 days (*n* = 43, 3, 26)	3.85 ± 0.75	3.65 ± 0.35	3.90 ± 0.58	0.218	0.804
HB (g/L)	≤28 days	124.50 ± 22.10	120.10 ± 18.10	120.35 ± 24.10	0.311	0.734
	>28 days	116.30 ± 21.50	113.10 ± 17.00	113.33 ± 23.50	0.220	0.803
HCT (%)	≤28 days	38.91 ± 7.10	38.10 ± 5.20	37.93 ± 7.40	0.521	0.596
	>28 days	36.00 ± 6.80	35.50 ± 5.00	34.70 ± 7.10	0.559	0.574
MCV (fL)	≤28 days	98.50 ± 9.10	97.50 ± 6.50	93.05 ± 10.10	1.889	0.158
	>28 days	91.80 ± 8.80	93.20 ± 6.20	88.20 ± 9.95^a^	1.810	0.170
MCH (pg)	≤28 days	31.10 ± 3.21	31.50 ± 2.20	30.01 ± 3.65	1.099	0.338
	>28 days	29.62 ± 3.10	29.75 ± 2.10	28.30 ± 3.55	1.082	0.344
MCHC (g/L)	≤28 days	321.35 ± 13.20	323.50 ± 5.60	323.83 ± 11.60	0.301	0.741
	>28 days	317.22 ± 13.10	319.00 ± 5.40	319.50 ± 11.35	0.311	0.734
RDW (%)	≤28 days	19.01 ± 11.01	19.10 ± 4.50	26.95 ± 15.10^a^	3.114	0.050
	>28 days	18.53 ± 10.80	18.00 ± 4.40	25.80 ± 14.85^a^	3.012	0.054

RBC, red blood cell; HB, hemoglobin; HCT, hematokrit; MCV, mean corpuscular volume; MCH, mean corpuscular volume; MCHC, mean corpuscular hemoglobin concentration; RDW, red cell distribution width.

^a^ Compared with the control group, *P* < 0.05.

^b^ Compared with the β-thalassemia moderate type group, *P* < 0.05.

### 3.3 Comparison of reticulocyte parameters between thalassemia patients and controls

The age-stratified analysis of reticulocyte parameters revealed that, in both age groups, the α-thalassemia group had significantly lower RET% and RET# compared to the control and β-thalassemia groups (*P* < 0.05). In contrast, the β-thalassemia group showed significantly higher IRF, MFR, and HFR compared to both the control and α-thalassemia groups, while LFR was significantly lower (*P* < 0.001) (Table 4). Subgroup analysis within the α-thalassemia cohort showed that RET% and RET# were significantly lower in both stationary and mild types compared to the control group in both age strata (*P* < 0.05) (Table 5). For β-thalassemia subgroups, there were statistically significant differences in IRF, LFR, MFR, and HFR among the control, β-moderate, and β-mild groups (*P* < 0.05), although pairwise comparisons did not consistently reveal significant differences between the β-thalassemia subgroups, again likely due to limited sample size (Table 6).

**Table 4:** Comparison of reticulocyte parameters among different thalassemia phenotypes and control groups, stratified by age.

Index	Age Group	Control group	α-thalassemia group	β-thalassemia group	F	*P*
RET%	≤28 days (*n* = 40, 85, 26)	1.65 ± 0.85	1.05 ± 0.50^a^	1.75 ± 0.68^b^	25.118	<0.001
	>28 days (*n* = 43, 92, 29)	1.23 ± 0.78	0.91 ± 0.45^a^	1.37 ± 0.62^b^	23.514	<0.001
RET# (10^9^/L)	≤28 days	65.10 ± 25.10	46.50 ± 14.80^a^	68.90 ± 25.50^b^	15.224	<0.001
	>28 days	44.80 ± 24.20	39.60 ± 14.30	53.10 ± 24.50^b^	14.662	<0.001
IRF (%)	≤28 days	18.90 ± 7.60	16.20 ± 6.60	24.50 ± 7.80^a^^,^^b^	17.112	<0.001
	>28 days	16.00 ± 7.50	15.40 ± 6.55	21.50 ± 7.70^a^^,^^b^	16.840	<0.001
LFR (%)	≤28 days	81.10 ± 7.60	83.80 ± 6.60	75.50 ± 7.80^a^^,^^b^	17.112	<0.001
	>28 days	84.00 ± 7.50	84.60 ± 6.55	78.50 ± 7.70^a^^,^^b^	16.840	<0.001
MFR (%)	≤28 days	13.80 ± 3.90	12.50 ± 3.70	16.90 ± 3.40^a^^,^^b^	17.553	<0.001
	>28 days	11.90 ± 3.80	12.00 ± 3.65	14.40 ± 3.25^a^^,^^b^	16.737	<0.001
HFR (%)	≤28 days	5.10 ± 4.30	3.70 ± 3.60	7.90 ± 5.30^a^^,^^b^	12.887	<0.001
	>28 days	4.10 ± 4.25	3.40 ± 3.45	6.85 ± 5.20^a^^,^^b^	12.364	<0.001

RET%, reticulocyte percentage; RET#, reticulocyte count; IRF, immature reticulocyte fraction; LFR, low fluorescence reticulocytes; MFR, medium fluorescence reticulocytes; HFR, high fluorescence reticulocytes.

^a^ Compared with the control group, *P* < 0.05.

^b^ Compared with the α-thalassemia group, *P* < 0.05.

**Table 5:** Comparison of reticulocyte parameters among different α-thalassemia subgroups and control group, stratified by age.

Index	Age Group	Control group	α-thalassemia silent type group	α-thalassemia mild type group	F	*P*
RET%	≤28 days (*n* = 40, 40, 45)	1.65 ± 0.85	1.15 ± 0.45^a^	0.96 ± 0.52^a^	9.881	<0.001
	>28 days (*n* = 43, 42, 49)	1.23 ± 0.78	0.98 ± 0.40^a^	0.85 ± 0.48^a^	8.756	<0.001
RET# (10^9^/L)	≤28 days	65.10 ± 25.10	48.10 ± 13.50^a^	45.05 ± 15.50^a^	6.553	0.002
	>28 days	44.80 ± 24.20	41.20 ± 13.30	37.50 ± 14.95^a^	5.855	0.004
IRF (%)	≤28 days	18.90 ± 7.60	16.10 ± 6.50	16.30 ± 6.30	1.011	0.367
	>28 days	16.00 ± 7.50	15.05 ± 6.35	15.15 ± 6.25	0.916	0.403
LFR (%)	≤28 days	81.10 ± 7.60	83.90 ± 6.50	83.70 ± 6.30	1.011	0.367
	>28 days	84.00 ± 7.50	84.95 ± 6.35	84.85 ± 6.25	0.916	0.403
MFR (%)	≤28 days	13.80 ± 3.90	12.20 ± 3.50	12.80 ± 3.85	0.712	0.493
	>28 days	11.90 ± 3.80	11.60 ± 3.40	12.10 ± 3.75	0.572	0.566
HFR (%)	≤28 days	5.10 ± 4.30	3.90 ± 3.60	3.50 ± 3.10	1.355	0.262
	>28 days	4.10 ± 4.25	3.45 ± 3.50	3.10 ± 3.00	1.239	0.293

RET%, reticulocyte percentage; RET#, reticulocyte count; IRF, immature reticulocyte fraction; LFR, low fluorescence reticulocytes; MFR, medium fluorescence reticulocytes; HFR, high fluorescence reticulocytes.

^a^ Compared with the control group, *P* < 0.05.

**Table 6:** Comparison of reticulocyte parameters among different β-thalassemia subgroups and control group, stratified by age.

Index	Age Group	Control group	β-thalassemia moderate type group	β-thalassemia mild type group	F	*P*
RET%	≤28 days (*n* = 40, 3, 23)	1.65 ± 0.85	1.25 ± 0.60	1.80 ± 0.65	0.911	0.406
	>28 days (*n* = 43, 3, 26)	1.23 ± 0.78	0.95 ± 0.58	1.42 ± 0.61	0.887	0.416
RET# (10^9^/L)	≤28 days	65.10 ± 25.10	45.50 ± 19.00	71.50 ± 25.10	1.755	0.180
	>28 days	44.80 ± 24.20	35.80 ± 18.00	55.20 ± 24.50	1.636	0.201
IRF (%)	≤28 days	18.90 ± 7.60	21.50 ± 9.20	24.80 ± 7.60^a^	4.772	0.011
	>28 days	16.00 ± 7.50	19.00 ± 9.10	21.80 ± 7.55^a^	4.328	0.016
LFR (%)	≤28 days	81.10 ± 7.60	78.50 ± 9.20	75.20 ± 7.60^a^	4.772	0.011
	>28 days	84.00 ± 7.50	81.00 ± 9.10	78.20 ± 7.55^a^	4.328	0.016
MFR (%)	≤28 days	13.80 ± 3.90	15.10 ± 3.40	17.20 ± 3.30^a^	5.221	0.007
	>28 days	11.90 ± 3.80	13.30 ± 3.35	14.50 ± 3.25^a^	4.871	0.010
HFR (%)	≤28 days	5.10 ± 4.30	6.40 ± 6.50	8.20 ± 5.20^a^	3.114	0.050
	>28 days	4.10 ± 4.25	5.70 ± 6.40	7.00 ± 5.10^a^	2.823	0.065

RET%, reticulocyte percentage; RET#, reticulocyte count; IRF, immature reticulocyte fraction; LFR, low fluorescence reticulocytes; MFR, medium fluorescence reticulocytes; HFR, high fluorescence reticulocytes.

^a^ Compared with the control group, *P* < 0.05.

### 3.4 Logistic regression model for predicting and differentiating α and β thalassemia

Through stepwise regression analysis, HCT, MCH, and RET% were selected as independent variables for the predictive model of α-thalassemia. The resulting formula was:

α-thalassemia prediction model: 9.604 + 4.374 × HCT−0.333 × MCH−0.803 × RET%

The receiver operating characteristic (ROC) curve was plotted to assess the model's performance. The area under the curve (AUC) was 0.815, with a cutoff value of 0.57, which corresponded to a sensitivity of 0.847 and a specificity of 0.675.

For the β-thalassemia prediction model, MCV, RDW, and MFR were selected through stepwise regression analysis. The resulting formula was:

β-thalassemia prediction model: −1.37 + (−0.045 × MCV) + 0.058 × RDW + 0.273 × MFR

This model exhibited an AUC of 0.786, with a cutoff value of 0.37, yielding a sensitivity of 0.782 and specificity of 0.675.

For differentiating between α-thalassemia and β-thalassemia, RBC, MCHC, RET#, and IRF were selected through stepwise regression analysis. The resulting discriminative formula was:

α vs. β-thalassemia discriminative model: −31.3−0.905 × RBC + 0.096 × MCHC + 0.035 × RET# + 0.094 × IRF

This combined discriminative model showed an AUC of 0.879, with a cutoff value of 0.36, corresponding to a sensitivity of 0.727 and specificity of 0.892.

**Figure 1:**
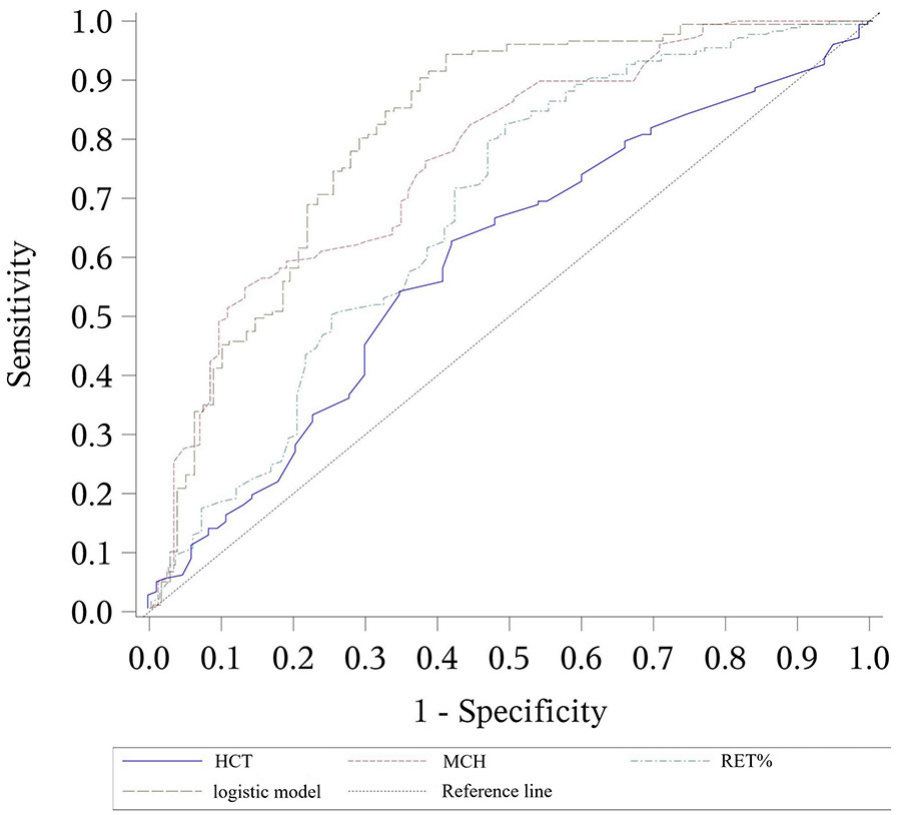
ROC curves for the logistic model predicting α-thalassemia, including the values of each parameter. The combined logistic model achieved an AUC of 0.815 (95% CI: 0.755–0.875) with an optimal cutoff value of 0.57. HCT, hematocrit; MCH, mean corpuscular hemoglobin; RET%, reticulocyte percentage.

**Figure 2:**
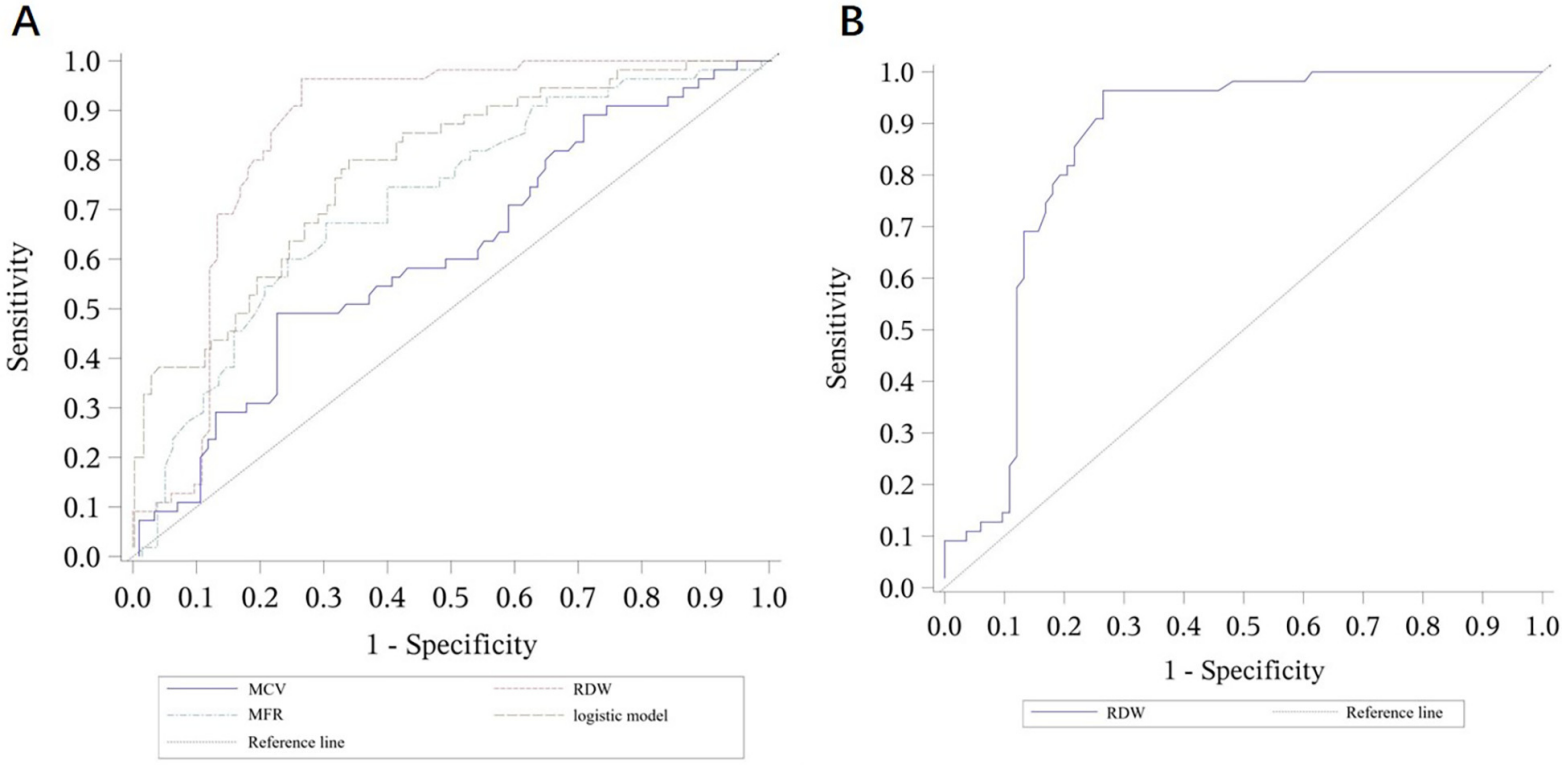
ROC curves for the logistic model predicting β-thalassemia and for the prediction model using RDW alone. **(A)** ROC curves for the logistic model predicting β-thalassemia, including the values of each parameter. The combined logistic model achieved an AUC of 0.786 (95% CI: 0.710–0.862) with an optimal cutoff value of 0.37. **(B)** ROC curves for the prediction model using RDW alone as a parameter. RDW alone achieved an AUC of 0.853 (95% CI: 0.787–0.920) with an optimal cutoff value of 16.35. MCV, mean corpuscular volume; RDW, red cell distribution width; MFR, medium fluorescence reticulocytes.

**Figure 3:**
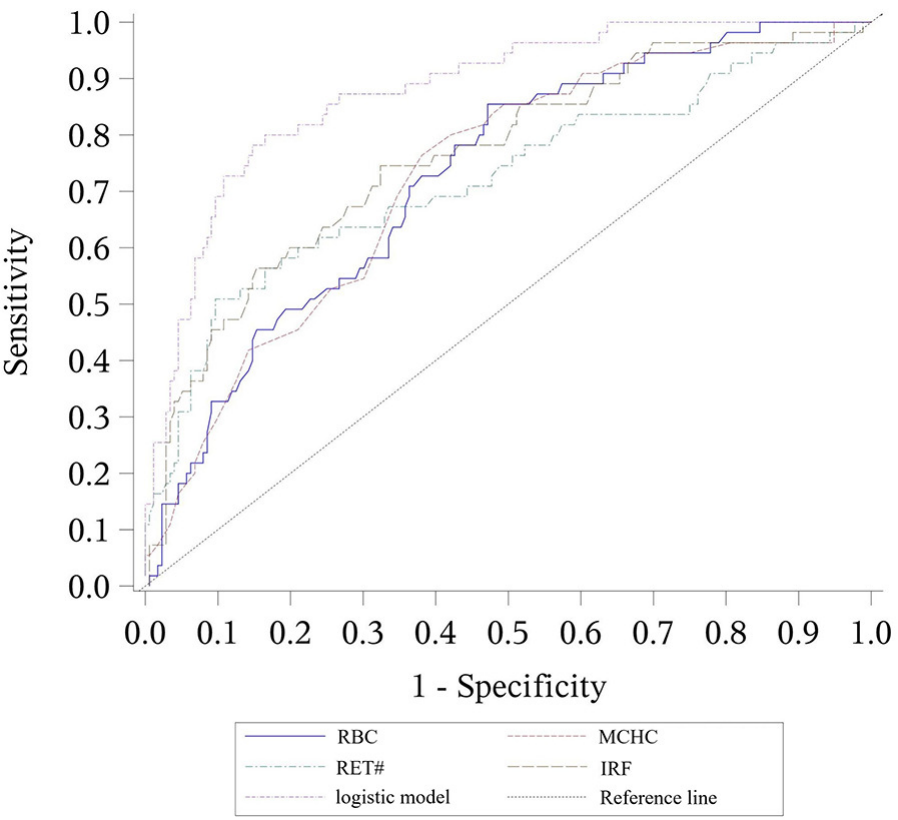
ROC curves for the logistic model discriminating between α-thalassemia and β-thalassemia, including the values of each parameter. The combined logistic model achieved an AUC of 0.879 (95% CI: 0.829–0.929) with an optimal cutoff value of 0.36. RBC, red blood cell count; MCHC, mean corpuscular hemoglobin concentration; RET#, reticulocyte count; IRF, immature reticulocyte fraction.

The accuracy of the combined logistic models in predicting α-thalassemia and differentiating between α and β-thalassemia was higher than that achieved by using a single parameter. However, the AUC for the β-thalassemia predictive model was lower than that of the prediction model using RDW alone as a parameter (Tables 7–9 and Figures 1–3).

**Table 7:** The ROC curves for the logistic model predicting α-thalassemia along with the values of each parameter.

Index	AUC	SE	Wald 95% CI	Cutoff	Sensitivity	Specificity
Logistic model	0.815	0.031	0.755–0.875	0.57	0.847	0.675
HCT	0.593	0.038	0.519–0.668	0.37	0.627	0.578
MCH	0.766	0.032	0.704–0.828	27.25	0.548	0.867
RET%	0.678	0.038	0.604–0.753	1.27	0.825	0.506
Reference line	0.500	0.000	0.500–0.500	-	-	-

HCT, hematokrit; MCH, mean corpuscular hemoglobin; RET%, reticulocyte percentage; AUC, area under the curve; SE, standard error; 95% CI, 95% confidence interval.

**Table 8:** The ROC curves for the logistic model predicting β-thalassemia along with the values of each parameter.

Index	AUC	SE	Wald 95% CI	Cutoff	Sensitivity	Specificity
Logistic model	0.786	0.039	0.710–0.862	0.37	0.782	0.675
MCV	0.609	0.049	0.512–0.705	89.85	0.491	0.771
RDW	0.853	0.034	0.787–0.920	16.35	0.964	0.735
MFR	0.717	0.044	0.630–0.804	15.55	0.600	0.759
Reference line	0.500	0.000	0.500–0.500	-	-	-

MCV, mean corpuscular volume; RDW, red cell distribution width; MFR, medium fluorescence reticulocytes; AUC, area under the curve; SE, standard error; 95% CI, 95% confidence interval.

**Table 9:** The ROC curves for the logistic model discriminating between α and β-thalassemia along with the values of each parameter.

Index	AUC	SE	Wald 95% CI	Cutoff	Sensitivity	Specificity
Logistic model	0.879	0.026	0.829–0.929	0.36	0.727	0.892
RBC	0.727	0.037	0.655–0.800	4.5	0.855	0.531
MCHC	0.726	0.038	0.652–0.800	312.5	0.855	0.506
RET#	0.722	0.044	0.637–0.808	61.5	0.509	0.903
IRF	0.762	0.038	0.687–0.836	21.9	0.564	0.847
Reference line	0.500	0.000	0.500–0.500	-	-	-

RBC, red blood cell; MCHC, mean corpuscular hemoglobin concentration; RET#, reticulocyte count; IRF, immature reticulocyte fraction; AUC, area under the curve; SE, standard error; 95% CI, 95% confidence interval.

## 4 Discussion

Thalassemia is an autosomal recessive inherited disorder that primarily affects the synthesis and function of hemoglobin. As a result, hematological abnormalities are commonly observed in affected individuals. Blood routine examination often reveals abnormalities in red blood cell parameters, such as reduced hemoglobin levels, decreased red blood cell count, and altered red blood cell morphology (12, 13). Due to the mild or asymptomatic nature of thalassemia in carriers with the mild or stationary forms, these individuals are frequently overlooked, contributing to a higher incidence of thalassemia. Therefore, implementing effective hematological screening methods and conducting screening programs for newborn populations are essential for early diagnosis and the development of prevention and control measures.

The study of hematological indicators plays a crucial role in the diagnosis and differentiation of thalassemia. Our study revealed that in the thalassemia group, MCV, MCH, and MCHC were decreased, with the α-thalassemia group exhibiting a more significant decrease than the β-thalassemia group. This finding was consistent across both neonatal age groups (≤28 days and >28 days), highlighting the robustness of these markers for early screening. In relation to the number of gene deletions, the α-thalassemia mild type group, which had more gene deletions, showed a more pronounced reduction compared to the α-thalassemia stationary type group. Conversely, the RBC count was higher in the α-thalassemia mild type group, with the highest count observed in this group, surpassing both the β-thalassemia group and the control group. The RDW was highest in the β-thalassemia group, and the β-thalassemia mild group had a higher RDW than the β-thalassemia moderate group, suggesting that genetic defects increase red blood cell variability. While the α-thalassemia group exhibited significant differences in RBC, MCV, MCH, and MCHC, the β-thalassemia group did not show major alterations in routine blood parameters but had a markedly increased RDW. Previous studies have shown that MCV, MCH, MCHC, and RDW, as common blood routine examination parameters, are highly valuable for screening thalassemia and assessing its clinical phenotypes (14, 15). The findings of our study are consistent with these previous reports.

Reticulocytes are important indicators of bone marrow hematopoiesis in thalassemia patients (16). The reduced RBC count leads to compensatory red blood cell production, which in turn stimulates robust bone marrow hematopoiesis and the premature release of immature reticulocyte fractions (IRF) into the peripheral blood. This results in increased HFR (high fluorescence reticulocytes) and a decrease in LFR (low fluorescence reticulocytes), ultimately raising the overall number of reticulocytes (RET#) and their percentage (RET%). The clinical severity of different types of thalassemia varies, which is reflected in the corresponding changes in reticulocyte parameters. As the disease progresses and bone marrow hematopoiesis enters a decompensated phase, both the total number and percentage of reticulocytes decrease, accompanied by more pronounced reductions in MCV, MCH, and MCHC. Schoorl (17) and Sun (18) have pointed out that in β-thalassemia patients, changes in routine blood parameters such as MCV and RDW-CV are minimal, making it insufficient to rely solely on MCV and RDW-CV for screening and differential diagnosis. Thus, simultaneous measurement of reticulocyte parameters is more meaningful. Our study also measured reticulocyte levels, revealing that the RET# and RET% in the α-thalassemia group were both lower than those in the control group and the β-thalassemia group, with the α-thalassemia mild type group (which had more gene deletions) showing the lowest values. In contrast, in the β-thalassemia group, while LFR was lower than in both the α-thalassemia and control groups, IRF, MFR, and HFR were all higher than in the other two groups. These findings confirm the reports in the existing literature and provide further support for these observations.

To further explore the role of common hematological indicators in predicting and differentiating various types of thalassemia, we constructed logistic regression models, using different parameters as independent variables. The results were consistent with our expectations: the combined detection models demonstrated higher accuracy in predicting α-thalassemia and differentiating between α- and β-thalassemia compared to single indicators. However, it was notable that the AUC of the combined detection model for predicting β-thalassemia was lower than that achieved using RDW alone as a diagnostic parameter. We speculate that this result may be due to the relatively small sample size, which could introduce bias. Furthermore, this outcome indirectly highlights the significant value of RDW, a parameter reflecting the uniformity of red blood cell size, in predicting β-thalassemia, as it showed high sensitivity. This aligns with research by Liu (19) and Hu (20).

Despite the strengths of this study, there are several limitations. Firstly, the sample size was relatively small, which may not fully represent the broader population, potentially introducing biases in the statistical results. Secondly, since all samples were from the Huangshi area, the findings may have limited generalizability, as thalassemia incidence and genotypes can vary across different regions and ethnicities. The prevalence and specific genotypes of thalassemia can vary significantly across different geographic regions and ethnic populations, which may influence the performance of our diagnostic models in other settings. Furthermore, the study faced a marked imbalance in subgroup sizes (e.g., β-thalassemia moderate type vs. mild type), which reflects the natural distribution of these genotypes but may affect the statistical power and reliability of subgroup comparisons. The use of stepwise regression for model building carries a risk of overfitting; future studies should employ more robust validation techniques, such as k-fold cross-validation or bootstrapping, to confirm the stability and generalizability of the predictive models (21). Additionally, the study did not include other important discriminative markers such as HbA2 or serum ferritin. Although challenging to interpret in the neonatal period due to physiological fluctuations, their exclusion limits the differential diagnostic scope, and they should be considered in future, more comprehensive follow-up studies. Due to funding and time constraints, the number of parameters included in this study was limited. Future research will aim to conduct multicenter studies, expand the sample size, incorporate more parameters, and refine the combined detection models to provide more accurate and comprehensive predictions for differentiating various types of thalassemia.

## 5 Conclusion

α-thalassemia is characterized by a significant decrease in MCV, MCH, MCHC, RET#, and RET%, along with a notable increase in RBC count, with the mild type exhibiting more pronounced changes than the stationary type. In contrast, β-thalassemia is characterized by a significant increase in RDW, IRF, MFR, and HFR, along with a marked decrease in LFR. MCV, MCH, and RDW can be used as initial screening indicators for thalassemia, and patients with clinically low MCV and MCH but high RDW should be referred for further genetic testing. The combined detection of blood routine examination and reticulocyte parameters may help improve the screening rate and differential diagnosis of thalassemia, making it a valuable approach for large-scale population screening to facilitate early diagnosis and treatment of thalassemia.

## Data Availability

The original contributions presented in the study are included in the article/Supplementary Material, further inquiries can be directed to the corresponding authors.
